# Mechanical and Tribological Performances of Thermoplastic Polymers Reinforced with Glass Fibres at Variable Fibre Volume Fractions

**DOI:** 10.3390/polym15030694

**Published:** 2023-01-30

**Authors:** Moustafa Mahmoud Yousry Zaghloul, Karen Steel, Martin Veidt, Michael T. Heitzmann

**Affiliations:** 1 School of Mechanical and Mining Engineering, The University of Queensland, Brisbane 4072, Australia; 2 Centre for Advanced Materials Processing and Manufacturing (AMPAM), The University of Queensland, Brisbane 4072, Australia; 3 School of Chemical Engineering, The University of Queensland, Brisbane 4072, Australia

**Keywords:** polyamide, man-made fibres, thermoplastic composites, wear performance, fibre volume fraction, wear behaviour, polymer composites, mechanical properties

## Abstract

High wear rates and frictional coefficients have always been the primary reasons for limiting the service life of critical elements such as pumps, couplings, bushings, bearings and gears. The premature and erratic failures are costing the industries extensive amounts of money every year. Additionally, under severe service conditions, the wear resistance requirements are higher, which greatly hinders the application of neat thermoplastics in different sectors. Hence, it is vital to enhance the tribological characteristics of thermoplastics. The mechanical and tribological properties of Polyamide 6, Thermoplastic Polyurethane, and glass fibre reinforced (GFR) Polyadmide 6 Composites of variable fibre volume fractions were investigated. Pin specimens of Polyamide 6 reinforced with (25%, 33%, and 50%) by volume of fibres were fabricated by an injection moulding process. The specimens were tested for tensile, compression, hardness, and wear under dry abrasive conditions using a pin-on-disc setup. Furthermore, the samples were scanned using micro-computed tomography (micro-CT), and the worn-out samples were analysed using field emission scanning electron microscopy. The experimental results showed that the fibre volume fraction was inversely proportional to the wear resistance of the prepared composite materials. This research will enable the industry partners to supply cutting-edge technologies to the global oil and gas industry that not only minimizes the well running cost but also improves the well resilience.

## 1. Introduction

The industry is seeing an unprecedented diversity of wear-related problems. The substitutions of mechanical components are the main drawbacks encountered by industries that consume excessive time and money. These replacements mainly occur due to wear, friction, fatigue, and corrosion. As a consequence, it is becoming increasingly vital to assess the wear behaviour and select an adequate material to guarantee sufficient life of the mechanical parts in order to minimise downtime and associated environmental impacts [1].

The wear mechanisms encountered by mechanical parts in the slurry environment are not trivial, and consist of complex three-body wear interactions between the type of the material, the type of counter-face material, and the sediment-rich transport fluid (i.e., slurry). There are a number of important factors that influence the wear rate, including the composition/properties of the transport fluid, service temperature, and contact pressure between the materials. The lack of reliable wear data is preventing industries from predicting and managing wear in the design and operational phases. The complexity of polymer tribo-systems makes them a multidisciplinary field of interest in many areas of application [2,3]. A great deal of research has been dedicated to understanding the various sliding and wear mechanisms of polymers in order to develop a clearer outlook of polymer tribology [4]. The wear mechanisms that have mostly received attention are the adhesive and abrasive wear types. However, polymers are not only subject to adhesive and abrasive wear but to a wide variety of other wear mechanisms, namely: thermal wear, corrosive wear, oxidation wear, chemical wear, surface fatigue wear, cavitation wear, erosive wear, debris wear, and even other types of wear like delamination, diffusion wear, solubilisation wear, and impact wear [5]. In fact, the actual characteristics of polymer wear depend on the dynamics/conditions imposed by the system. This complex interaction with the system and the environment results in a complex and diverse wear response. In addition, the selection of modern plastics and polymer composites available as wear liners is enormous and ranges from soft elastomers to high stiffness and hardness engineering thermoplastics and composites [6,7,8]. The associated ease of processing and mechanisms of polymer wear across these systems are correspondingly diverse [5]. For example, the wear may be described in one case by the formation of a transfer film; in a second case by the melting of its surface; and in a third case, by elastic or viscoelastic deformation at the contact region. A combination and compounding of simultaneous mechanisms are also possible.

Thermoplastic composite materials have gained more and more scientific interest owing to their excellent fatigue resistance, high specific strength, high specific modulus, and, specifically, their high tribological characteristics, and they have been primarily utilised in automobile, aerospace manufacturing, and industrial sectors [9,10,11,12]. Commonly, the applications in extremely abrasive applications include conveyor belts, bearings, vanes, bushes, seals, wind blades, bushings, gears, and pumps transferring industrial fluids [1]. Recently, several research investigations have focused on enhancing the mechanical behaviour of thermoplastic materials by incorporating inorganic and organic nano additives, such as carbon-based materials, polytetrafluoroethylene (PTFE), molybdenum disulphide (MoS_2_), and titanium dioxide (TiO_2_) [13,14,15,16]. The thermoplastic tribo-composite materials can be altered by adding an adequate amount of reinforcements, including aramid fibre, carbon fibre, glass fibre, as well as solid lubricants, which can boost the wear performance for the anticipated wear situations or modes [17,18,19,20,21,22,23,24].

Polyamide (PA), as a wear-resistant engineering thermoplastic, possesses self-lubricating properties, high wear resistance, and superior tensile strength because of the existence of hydrogen bonds and van der Waals forces in the molecular chain structure of polyamide. Hence, polyamide is favourable for use in the manufacturing of several engineering components that undergo wear and friction, including bearing and gears [25,26]. The improvement of tribological and mechanical characteristics of PA is possible by incorporating solid lubricants and fibres [27,28,29]. The addition of short carbon fibre (CF) at three fibre volume fractions: 10%, 20%, and 30% to a series of polyamide 6 (PA6) was tribo-tested under dry sliding conditions, and the optimum wear resistance (WR) was observed in the case of PA6 reinforced with 20% by volume of carbon fibres [30]. The scientific studies reported that the frictional coefficient could, generally, be minimised and the wear performance of thermoplastics sliding against steel gets enhanced when the thermoplastics are reinforced with aramid, carbon, or glass fibres [31,32]. The tribological performance is, nevertheless, affected by several material parameters, such as fibre volume fraction, fibre type, fibre shape, fibre orientation, matrix composition, and loading conditions, such as speed, load, distance, temperature, humidity, and counter-face roughness [33].

**Figure 1:**
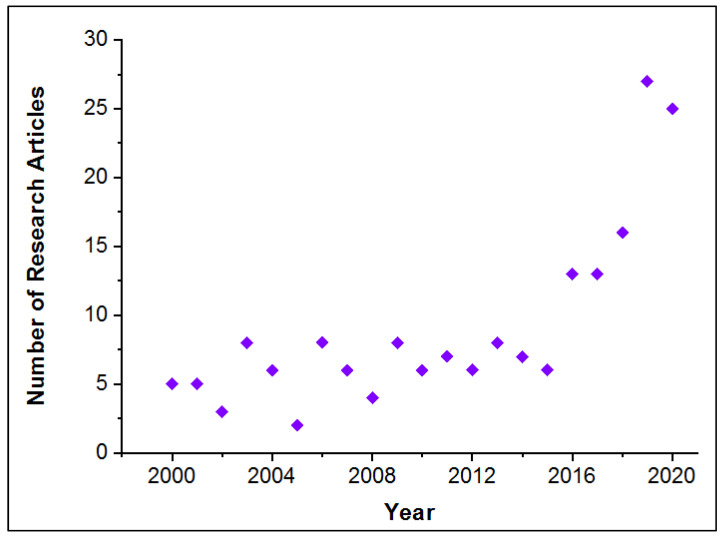
Research articles within the last twenty years in wear of varied fibre volumetric fractions (Two keywords used: “fibre volume fraction” and “wear”—Database used: www.sciencedirect.com, accessed on 5 December 2022).

Figure 1 represents the number of research papers in the last twenty years that studied the wear of materials at variable fibre volumetric fractions. The fewer studies in the previous years were a main drive to assess the wear performance of reinforced polymers by varying the fibre volume fractions. In light of the research gaps identified in the review article by Moustafa et al. [1], the current study aims to investigate the influence of fibre volumetric fraction on the abrasive wear performance of polymeric materials. The paper is presented in five sections; apart from the introduction provided in Section 1, Section 2 displays materials and methods followed, Section 3 shows the results in the form of figures and tables, and Section 4 the discussion of obtained results. Finally, the conclusion, along with closing remarks, appears in Section 5. In our present study, polyamide reinforced with glass fibres was prepared via the injection moulding method. Different volume fractions of glass fibres were added to the two selected polymers. The mechanical properties of the prepared compositions were investigated, including wear, tension, compression, and hardness. The physical and microstructural behaviours were studied in detail. Last but not least, the micro-CT analyses of all prepared samples were conducted to assess the orientation of fibres and their quantification.

## 2. Material and Methods

In this study, commercial polymeric composite materials were selected, which were marketed as compounds with excellent wear performance. The selected polymers for the study allow the establishment of basic trends such as the influence of fibre volume fraction, polymer type, and lubricant. The objective of this study is to set a benchmark on wear performance of current ‘best in class’ commercial compounds. This study will provide a first insight into key aspects of wear and find out the matrix material at which the wear resistance property is the highest. The list of raw materials and the preparation technique are introduced in this section.

### 2.1. Raw Materials

The matrix materials are PA6 and TPU. At the same time, the reinforcement type used in all polymers is glass fibre. Furthermore, Molybdenum disulphide (MoS_2_), as a lubricant, was used to check its effect on the wear performance of polymeric composite materials. Table 1 presents the physical properties (density, thermal conductivity, coefficient of friction, and tensile strength) of the two polymeric materials used in this first research study.

### 2.2. Preparation

The pellets of the pure thermoplastics and glass fibre-reinforced thermoplastics used were pre-dried. The pre-dried pellets of the designed formations were extruded by Duromer, Australia, and the injection moulding of the thermoplastic polymers and their composites was carried out using the injection moulding machine HXF 168 by NINGBO HAIXING PLASTICS MACHINERY, China. The moulds utilised provided samples for tensile, compression, hardness, and wear testing. The list of the thermoplastics used and their chemical composition is displayed in Table 2.

### 2.3. Mechanical Testing

This section describes the characterisation techniques carried out to assess polymeric composite materials. It presents the details of the physical, mechanical, and micro-structural characterisations of the prepared unreinforced polymeric and glass fibre-reinforced polymeric composite materials.

#### 2.3.1. Tensile Testing

**Figure 2:**
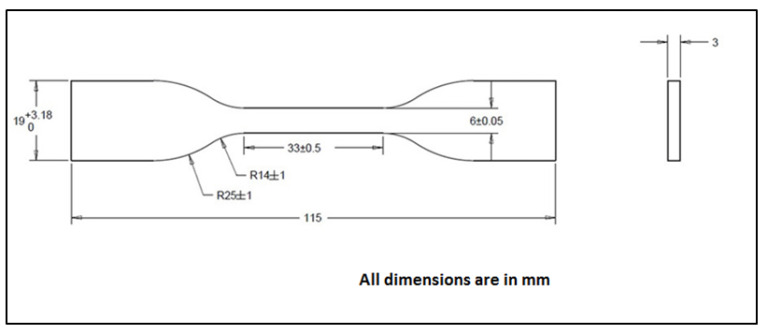
Tensile specimen geometry and dimensions.

**Figure 3:**
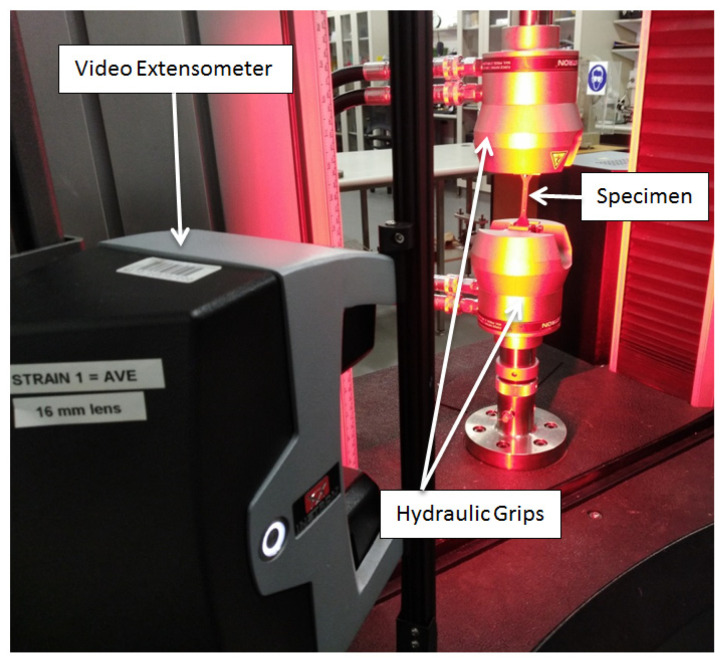
Tensile Testing of Specimens using INSTRON Universal Testing Machine.

The tensile testing of the neat polymers and reinforced polymers was performed by means of a universal testing machine supplied by INSTRON, Germany, model 5900R 5584. The maximum load capacity of the machine is 100 KN, while the load cell used for the testing of all specimens was 10 KN, and the samples were held and clamped by self-aligning hydraulic grips. The tensile tests were carried out at room temperature and 50% relative humidity. All surfaces of specimens were checked to ensure they were free of imperfections and flaws. Then, the samples were dried for 24 h prior to the test in a drying oven at 60 °C. The cured dog bone-shaped composite samples shown in Figure 2 were used for the tensile test as per ASTM D638-10 standard [34] in type IV specimen format. The gauge length and cross-head speed were set to 33 mm and 5 mm/min, respectively. Additionally, for the accuracy of recording displacements while excluding any possible slippage occurrences between the specimens and the grips, an extensometer was connected and set at the entire gauge length of the specimens in order to determine the live distance between two designated points on each specimen, while the specimen was being stretched. Furthermore, for the sake of verifying results, five tensile samples of each type of the prepared specimens were tested in accordance with the ASTM standard previously described. The results were utilised to evaluate the mean Young’s modulus and tensile strength of the specimens. The geometry of the tensile specimen and the universal testing machine used is displayed in Figure 2 and Figure 3, respectively.

#### 2.3.2. Compression Testing

The cured polymeric composite specimens of the required dimensions (length of 15 mm and diameter of 10 mm) were utilised for the uni-axial compressive testing as per the ASTM D 695 standard [35] for the compressive testing of rigid plastics. This helps to minimise buckling, reduce friction because of the small cross-sectional area, and avoid premature failure because of sharp corners. The static uni-axial compression tests were carried out on the specimens using Universal Testing Machine (INSTRON, Darmstadt, Germany, model 5900R 5584) at room temperature. Five identical samples were examined for each material design configuration, and the mean results of the compressive strength of the samples were recorded. The maximum compressive strength was calculated by dividing the highest compressive force endured by the sample during the test by the nominal cross-sectional area of the sample. The crosshead speed was maintained constantly at 1.3 mm/min, and the compression test was stopped when the sample showed failure signs or when 60% of the initial specimen length was reduced, whichever came first.

#### 2.3.3. Hardness Testing

Shore D hardness measurement was done using an INSTRON Shore D hardness tester and in accordance with the ASTM D2240-15 [36] standard. Shore D was selected as it applies to harder plastics and elastomer materials, while Shore A could be used for softer plastics. The scale range in Shore D hardness is 0 to 100. The low hardness number indicates a low hardness and vice versa. The specimen diameter and height were 10 mm and 15 mm, respectively. The samples were cleaned with alcohol before hardness testing. After samples were completely dried, the hardness test was carried out under ambient conditions, which were 25 °C and 50% relative humidity. The readings were recorded 1 s after the indentation was made. Ten hardness measurements were recorded per sample, and the mean values were calculated.

### 2.4. Wear Testing

**Figure 4:**
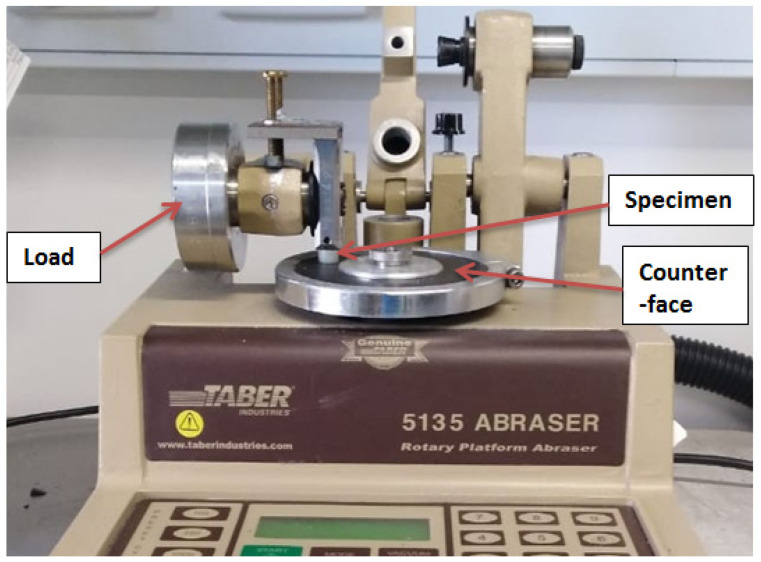
The testing setup using the tribometer apparatus, 5135 Abraser.

The tribological characteristics of the polymeric specimens were performed using the pin-on-disk wear testing machine, rotary platform abrader 5135, as shown in Figure 4.

The wear tests of specimens were carried out in accordance with ASTM G99-17 [37] under dry sliding conditions. The geometry of the pin specimens was cylindrical in shape, whose diameter and height was 10 mm and 15 mm, respectively. The surfaces of all polymeric specimens were uniformly polished by a polishing cloth covering a rotating disk in order to set a similar surface finish for all specimens.

Before testing, the samples were cleaned from dirt and foreign materials, followed by being dried at 50 °C for six hours. The dimensions of all specimens were measured using a Vernier Calliper with a scale value of 0.01 mm. The mass of specimens was measured using a balance with 0.0001 g sensitivity. The pin specimens were securely inserted in the sample holder, ensuring that the specimens were normal to the rotating disk when in contact in order to meet the required contact conditions.

The counter-face material in the wear tests was Silicon carbide (SiC) abrasive sandpaper at an outer diameter of ø102 mm and a grit number of 500. A new SiC abrasive sand paper was utilised for every wear test. The surface roughness parameters of the abrasive papers were measured using an optical profiler, Zeta 300. The surface roughness parameters for the papers were: R_a_ = 20.3 ± 0.12 μm and R_z_ = 81.2 ± 0.77 μm.

The applied sliding force and sliding velocity were set at 10 N and 72 RPM (0.31 m/s), respectively. The track radius was 41 mm in all wear tests, and the number of cycles was adjusted to keep the sliding distance at 257.48 m for all specimens. The wear test was carried out at room temperature (25 ± 2 °C) without interruptions. After each test was completed, the specimen was removed, cleaned of any wear debris using ethyl alcohol, and dried.

The mass losses of samples were calculated according to Equation (1) as follows:

∆m = m_i_ − m_f_ [mg] (1)

where ∆m is the mass loss of the sample, m_i_ is the initial mass of the sample, and m_f_ is the final mass of the sample after the wear test.

The density of the materials is calculated using a density kit and in accordance with the ASTM D792 standard for the density evaluation of plastics [38]. Five specimens per configuration were examined, and the average values of densities were recorded. The mass loss was converted into wear volume (∆V), which is the ratio between the mass loss and the calculated density of materials, as represented in Equation (2):

(2) $$\mathrm{Volumetric}\;\mathrm{loss}\;(\Delta V)=\frac{\mathrm{Mass}\;\mathrm{loss},\left(g\right)}{\mathrm{Density},\left(g.{\mathrm{cm}}^{-3}\right)}\;\times \;1000\;\;\;({\mathrm{mm}}^{3})$$

The calculated data from the wear test allows evaluation of the specific wear rate (W_s_) by the use of Equation (3), as follows:

W_s_ = ΔV/F_N_ L [mm^3^/N.m] (3)

where ΔV is the volume loss and L is the sliding distance.

### 2.5. X-ray Diffraction Analysis

The X-ray Diffraction (XRD) measurements were carried out on a Bruker (D8 Advance) with Cu Kα2 radiation in a range of 2θ = 10–60° using a fixed time mode with a step interval of 0.02° per minute and DIFFRAC.EVA Version 5.1 was used to set the amorphous and crystalline components of the materials in addition to measuring their crystallinity degree.

### 2.6. Micro-CT Analysis

The samples were prepared to be scanned with the SkyScan 1272 (Bruker, Kontich, Belgium) in order to assess the fibre orientation and distribution. The parameters used for acquisition include 1.25 μm voxel size, 70 kV voltage, 111 μA current, Al 0.5 mm filter, 2175 ms exposure time, 2 × 2 binding, frame averaging 2, and a 0.18-degree rotation step around 360 degrees of the sample. Micro-CT data were reconstructed with InstaRecon (InstaRecon, Urbana, IL, USA) & NRecon (Version 1.7.4.6, Bruker, Kontich, Belgium) using a back-projection algorithm with ring artefact reduction and correction for beam hardening. Data visualisation was generated using CTVox (Bruker, Kontich, Belgium).

### 2.7. Morphological Analysis

To further understand the wear forms after the abrasion tests, the worn-out surfaces of the test coupons were scanned by using the JEOL 7100 field emission scanning electron microscope (FESEM). It is well understood that FESEM is a vital tool in the tribological sector that assists in studying morphology on the sub-surface or interface of the materials as well as localising and analysing the formations of wear debris on the surfaces of materials. After tribological tests, the worn-out surfaces of the thermoplastic composite samples were plasma cleaned and finally coated with a conducting platinum layer of 20 nm thickness. The specimens were placed in a standard FESEM stub with two-sided carbon tape that faces the worn track/worn surface upwards. In FESEM, the operation parameters like the accelerating voltage in KV and probe current were optimised to achieve high-quality images without any artefacts such as surface degradation and sample charging. It was found that 10 KV and 8 A current showed the optimum scanned results. The images were recorded at various magnifications to obtain representative views of the worn-out surfaces.

## 3. Results and Discussion

In the following section, the mechanical characteristics of pure polymers and fibre-reinforced polymers are presented and discussed. The types of mechanical tests performed were: tensile testing, compression testing, hardness testing, and wear testing.

### 3.1. Tensile Behaviour of Materials

The average tensile modulus of PA6 and TPU were 2.2 GPa and 0.84 GPa, respectively. The yield strength and ultimate tensile strength of PA6 were higher than TPU by 117% and 148%, respectively.

**Figure 5:**
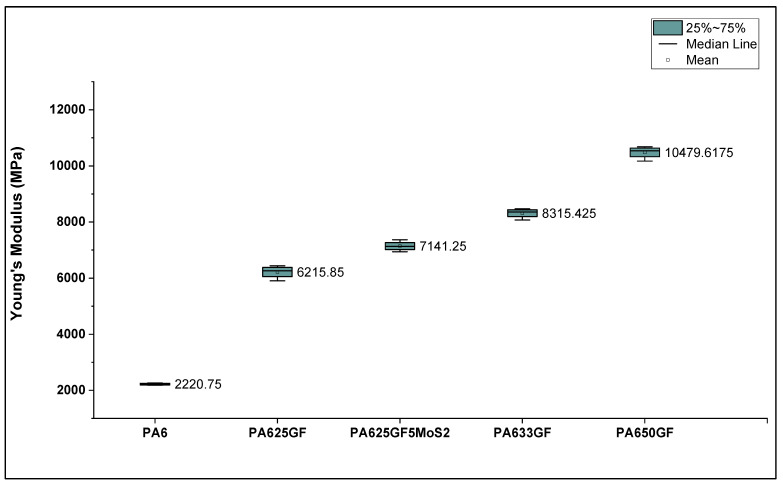
Elastic modulus for PA6 and PA6 reinforced with glass fibres at varied fibre volume fractions.

The tensile properties of PA6 and its composites (25%, 33%, and 50%) by volume of glass fibres are presented in Figure 5, which shows that PA650GF has the highest Young’s modulus, yield strength, and ultimate tensile strength. PA650GF is higher in yield strength than the least candidate material, PA6, by 231%. This result shows that the increase in fibre volume fraction is directly proportional to the stiffness, Young’s modulus, and ultimate tensile strength of the composite materials, which is also in agreement with the rule of mixtures theory. In addition, it was found that the addition of 5% MoS_2_ lubricant to PA625GF showed an average increase of 14% in Young’s modulus than without the lubricant.

### 3.2. Compressive Behaviour of Materials

**Figure 6:**
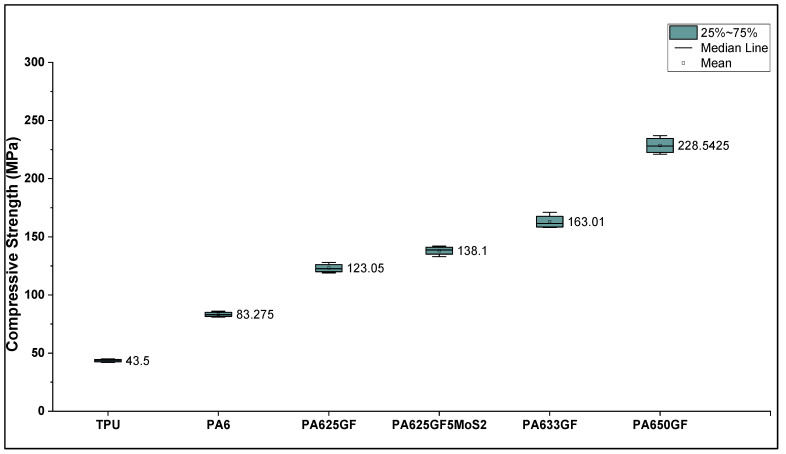
Compressive strength for polymers and their fibre-reinforced variations.

The compressive strength for the two polymer candidates and their reinforced variations is presented in Figure 6, which shows that PA6 possesses higher compressive strength than TPU by 91.43% at 30% strain. Moreover, for the glass fibre reinforced PA6, it can be observed that the increase in fibre volume fraction was directly proportional to the compressive strength values, whereas PA6 reinforced with 50% by volume of glass fibres was higher than that of PA633GF and PA625GF by 40% and 85.7%, respectively. The presence of lubricant slightly increased the compressive strength of PA6 by 11.7%, respectively. These results are in close agreement with the results obtained from the tensile testing of the materials, as described in the previous section.

### 3.3. Hardness Behaviour of Materials

**Figure 7:**
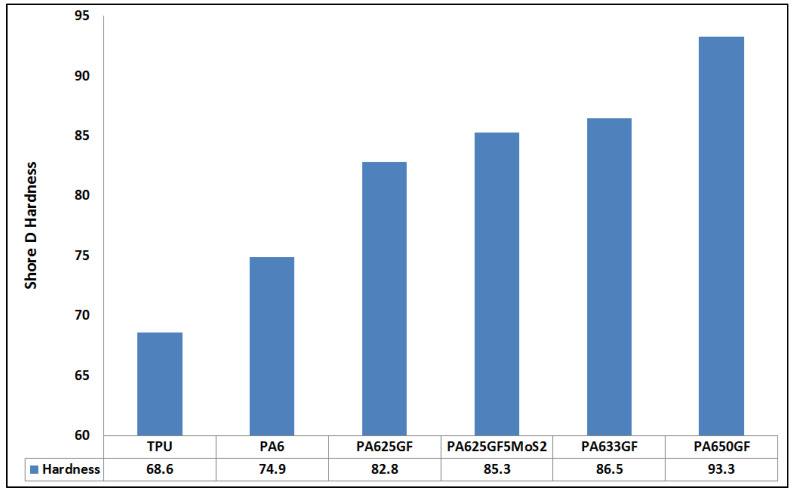
Shore D hardness values of the polymeric and composite samples.

The mean values of Shore D hardness readings of the specimens with different formulations are displayed in the bar chart in Figure 7. It is anticipated that the increase in the number of rigid components in a relatively softer component leads to a rise in the hardness values. Other elements that affect the values of hardness in fibre-reinforced thermoplastics are the fibre size, fibre type, fibre volume fraction as well as the adhesion strength between the matrix and the fibre. On account of this, the contribution of the fibre volume fraction was proved by the hardness testing results, as tabulated in Figure 7, which shows that the thermoplastic composite materials possessed higher hardness values than the plain polymers. The 50% filled glass fibre-reinforced thermoplastic materials had considerably higher hardness values, as high as 93.3 Shore D, than those of other thermoplastic materials because of the rigid structure of the fibres and their higher volumetric fraction.

### 3.4. Wear Behaviour of Materials

**Figure 8:**
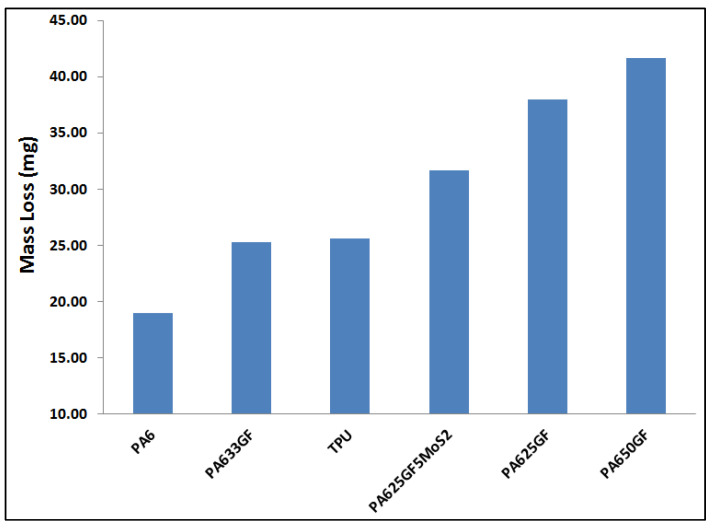
Mass loss values of the polymeric and composite samples after wear test.

The mass loss values of the pure polymers and their composites at different configurations against the Silicon carbide, grinding paper counter-face are given in Table 3 and Figure 8, respectively. In polymeric composite materials, the size, shape, and type of the additive material and chemical treatments present significant effects on the wear and mechanical characteristics of the composite materials. For example, short fibre lengths minimise the fibre load carrying capacity and lead to elevated wear rates, while long fibre lengths show a rise to the pull-out of the fibres and lead to excessive wear rates. Hence, utilising fibres smaller than the critical length was aimed at reducing stress transfer efficiency.

#### 3.4.1. Wear of Polymers against SiC Counter-Face

The wear rate of the two polymer candidate materials was assessed, and it was found that TPU exhibited a 32.73% higher wear rate than PA6, which had an average wear rate of 0.74 g/m × 10^−4^. This result indicates that the rise in temperature due to the action of the friction between the candidate materials and the counter-face led to a higher rate of material loss in TPU than that for PA6. The wear amount of polymers was measured by the loss in mass and volume. The mass loss was converted into wear volume (∆V), whereas five specimens per configuration were examined, and the average values of densities were recorded. Table 4 shows the calculated densities of the polymeric candidate materials and the wear amount in both volumetric loss and mass loss for each material.

Taking the wear volume into account and the applied sliding force, the specific wear rate of the polymeric materials was evaluated. The specific wear rate of PA6 had an average of 6.59 mm^3^/N.m × 10^−3^, which is 27.2% less than that of TPU.

#### 3.4.2. Wear of Polymeric Composite Materials against SiC Counter-Face

**Figure 9:**
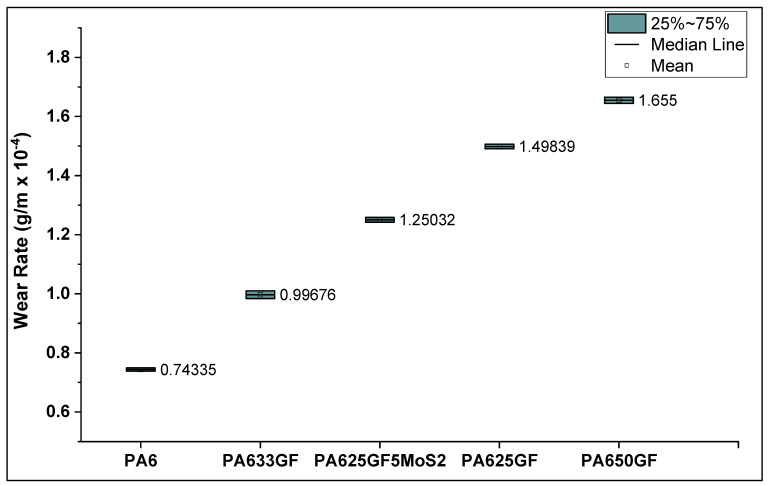
Wear rate of PA6 reinforced with glass fibres at varied fibre volume fractions.

The wear rate of PA6 reinforced with three different fibre volume fractions, which were (25%, 33%, and 50%) and PA6 reinforced with glass fibre at 25% fibre volume fraction in addition to 5% of the lubricant material MoS_2_ is represented in Figure 9, which shows that PA650GF exhibited the highest wear rate. The wear rate of PA650GF was 121.3% higher than that of pure PA6. Additionally, the role of the lubricant was significant in reducing the wear rate, where the addition of 5% of MoS2 led to a reduction in the wear rate by 19.87% when comparing the wear rates of PA625GF+5% MoS_2_ and PA625GF.

The lubricant was significant in minimising the wear at interfaces in relative motion, hence minimising the wear rate and increasing the lifetime of structures. It is generally understood that the thermal resistance of thermoplastic composite materials has a large influence on their wear and friction behaviours. The friction heat is generated in the frictional processes, particularly in the condition of dry abrasion, which cannot be resisted in the absence of a lubricating media such as water, oil, and other fluids. Therefore, thermoplastic composite materials become more prone to surface deterioration due to the presence of the generating frictional heat. Besides, thermoplastic composite materials with good thermal stabilities are not easy to get damaged by the effect of high temperature in the friction process. In our current research, the PA625GF+5% MoS_2_ composites possess good thermal stability, which is beneficial for their wear characteristics.

Additionally, PA625GF+5% MoS_2_ samples have higher hardness, which means that they have higher load-carrying capacities and can further protect PA6 against worn-off conditions. This enhancement of wear performance due to the addition of MoS_2_ is in good agreement with previous studies [39,40]. Furthermore, among the three fibre volume fractions used while reinforcing PA6, it was found that 33% of glass fibre content by volume was the optimum percentage in resisting wear, where the wear rate of PA633GF was less than these of PA625GF and PA650GF by 50.2% and 65.59%, respectively.

The wear amount of composite specimens was measured by the loss in mass and volume. The mass loss was converted into wear volume (∆V) whereas five specimens per configuration were examined, and the average values of densities were recorded. Table 5 shows the calculated densities of the fibre-reinforced composite materials and the wear amount in both volumetric loss and mass loss for each material.

**Figure 10:**
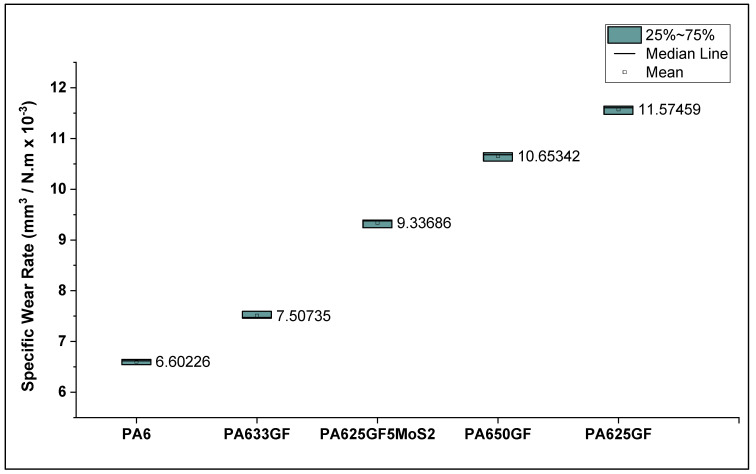
Specific wear rate of PA6 reinforced with glass fibres at varied fibre volume fractions.

Taking the wear volume into account and the applied sliding force, the specific wear rate of the polymeric materials was evaluated and presented in Figure 10. It can be deduced that the incorporation of glass fibres lowers the wear resistance of thermoplastic composite materials. Similar to what was observed through hardness testing, it can be observed in Figure 10 that the specific wear rate values were directly proportional to the fibre volume fraction in the thermoplastic composites. This result can be attributed to the rigidity of the structure at high fibre fractions, which led to increasing abrasive wear between the specimens and counter-faces. This result is consistent with the previous research findings reported in the literature survey, which report that the abrasive wear resistance of thermoplastic composite materials can be proportional to the rigidity or fibre volume fraction in the fibre-reinforced composite systems.

**Figure 11:**
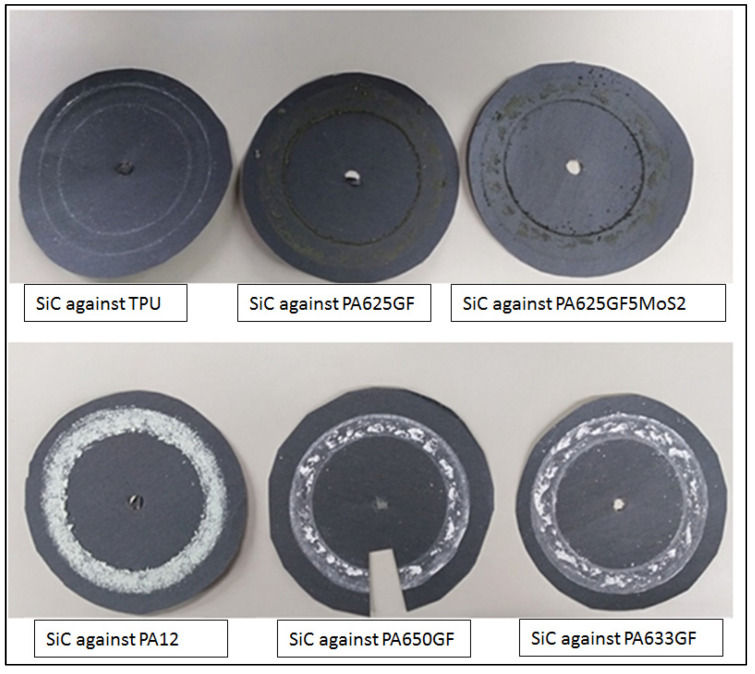
Wear track on SiC grinding papers counter-face after conducting the wear test.

The images of the worn surfaces of the counter-face materials after conducting the wear tests are shown in Figure 11. The worn-out surfaces of the counter-face materials were scanned using the optical profiler Zeta 300. The worn surfaces of the counter-face materials are relatively rough when compared to the unworn side surfaces of the SiC grinding papers due to abrasive wear. When the polymeric materials slide over the Silicon carbide counter-face grinding papers, the adhesion between the counter-face and the candidate polymers is of sufficient amount to inhibit sliding at the original interface. Hence, the junctions tear within the polymeric specimens, and a thin layer of each specimen was deposited on the counter-face material in the form of a coherent thin film located in the wear track. The subsequent motions of the polymeric specimen over this thin layer removed the transferred film, and a new layer was deposited. This process was repeated while the surfaces of the polymeric specimens were gradually worn away.

### 3.5. XRD Analysis of PA6 and PA6 Composites

**Figure 12:**
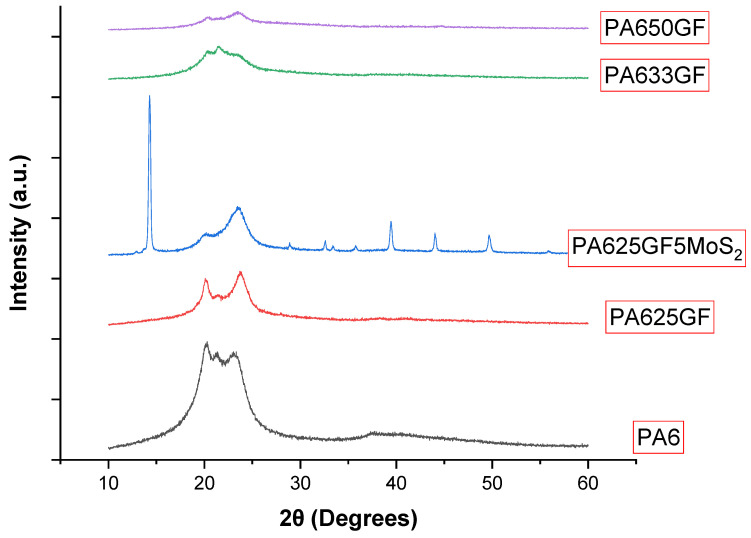
XRD patterns of PA6 and PA6 composite materials.

XRD analyses were carried out to identify the crystallographic nature of the PA6 and PA6 composite materials and the influence of this crystallinity degree on the wear performance of the studied materials. From the XRD results displayed in Figure 12, the peaks at 2θ = 20.8° (200) and 22.9° (202, 002) define the characteristic peaks of PA6.

The degree of crystallinity of the studied materials is displayed in Table 6. The addition of glass fibres led to a decrease in the intensity of the characteristic peaks. However, the addition of MoS_2_ improved the crystallinity of the materials remarkably from Xc = 40.52 to Xc = 52.38. This increase in the crystallinity degree corresponded to reduced specific wear rates.

### 3.6. Micro-CT Analysis of Materials

**Figure 13:**
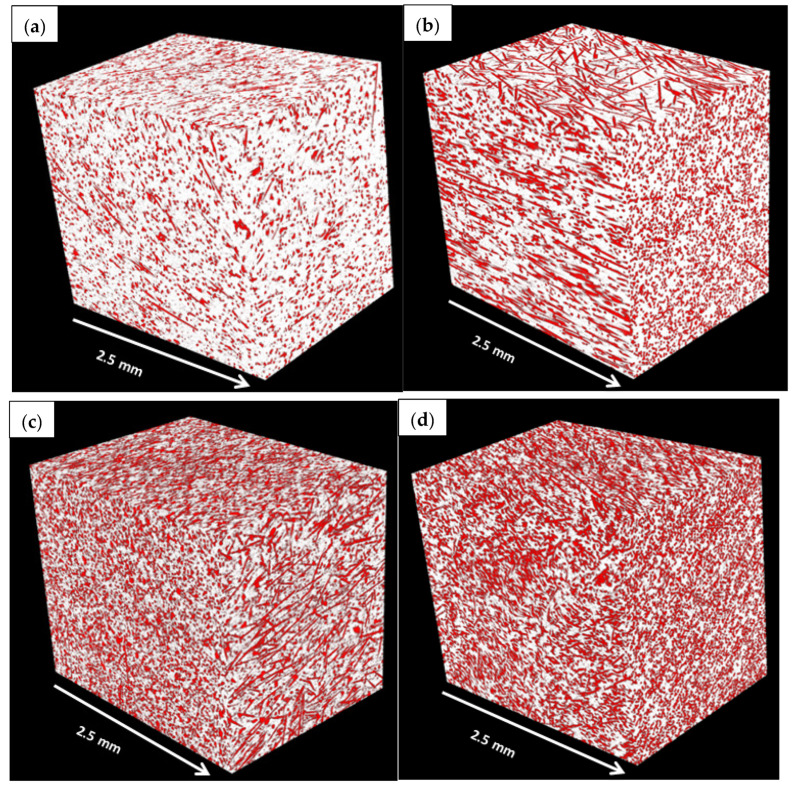
Tomogram images 3D visualisations of the central part of the plain thermoplastic samples; each subset represents roughly one-third of the samples’ volumes imaged; ((**a**): PA625GF, (**b**): PA625GF+5MoS_2_, (**c**): PA633GF, and (**d**): PA650GF).

The micro-CT was performed to investigate fibre distribution and alignment, as well as voids in the composites. TPU showed fewer impurities and fewer voids than PA6. On the other hand, the 3D tomogram images of the polymeric-reinforced samples are presented in Figure 13. For all fibre-reinforced samples, the fibres appear to be randomly distributed of different lengths. In addition, the fibre volume fraction could be verified that comes in line with the produced volumes. The red colour represents the fibre phase, while the white colour represents the matrix phase. It can be seen that uniform fibre distribution was detected without any fibre agglomerates. It was seen that the fibre alignment of the thermoplastic composite materials made by injection moulding was in the direction of the mould filling. Generally, thermoplastic composite materials made by injection techniques were closely packed, which resulted in negligible voids, as displayed in Figure 13.

### 3.7. Surface Morphology of Worn-Out Surfaces

**Figure 14:**
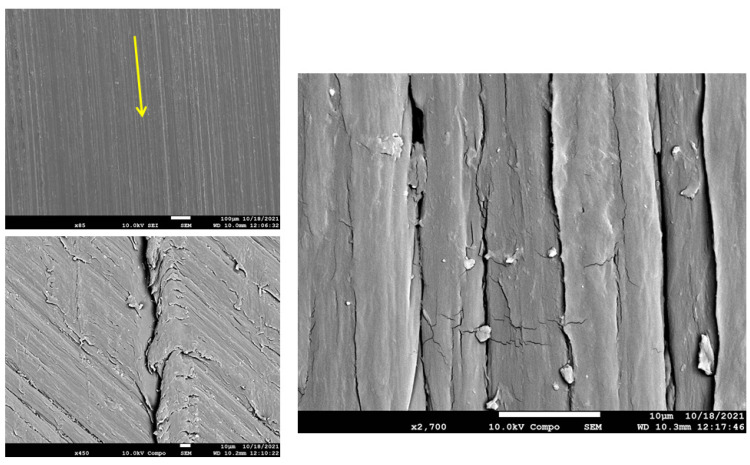
FESEM Micrographs for the worn-out surfaces of PA6 at different magnifications.

**Figure 15:**
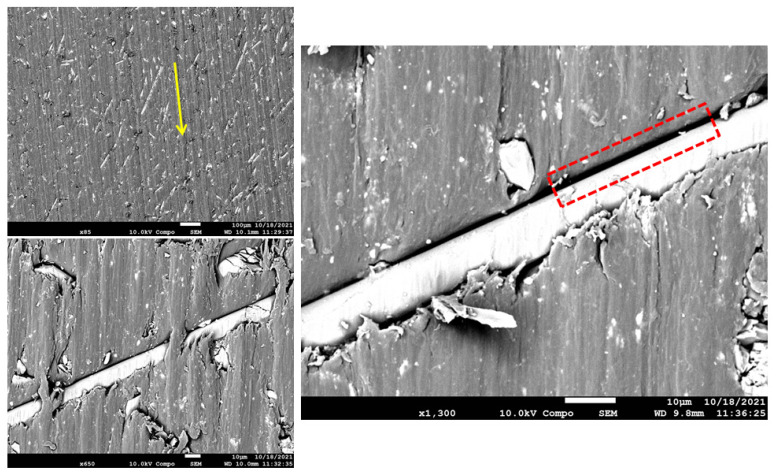
FESEM Micrographs for the worn-out surfaces of PA625GF at different magnifications.

**Figure 16:**
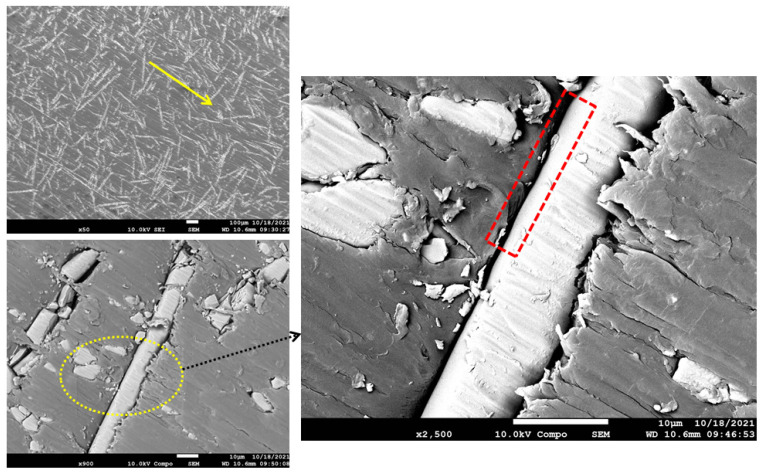
FESEM Micrographs for the worn-out surfaces of PA633GF at different magnifications.

**Figure 17:**
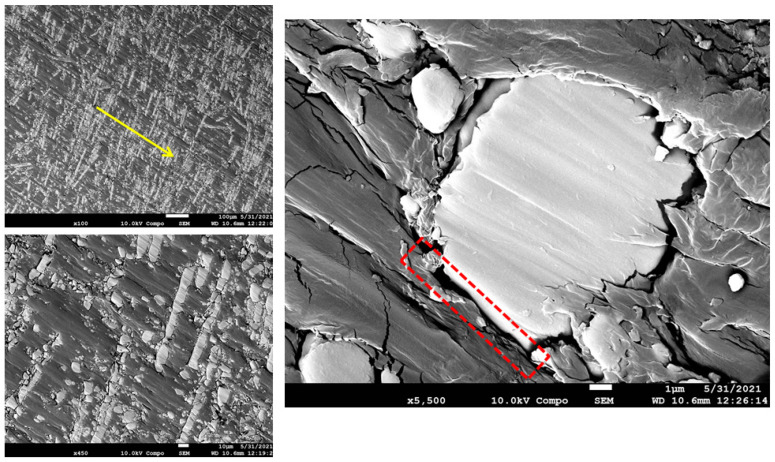
FESEM Micrographs for the worn-out surfaces of PA650GF at different magnifications.

The worn-out surface morphologies of the thermoplastic composite materials have been examined by field emission scanning electron microscopy (FESEM), and the micrographs of the worn surfaces are displayed in Figure 14, Figure 15, Figure 16 and Figure 17. As a general observation from all micrographs, there were no noticeable fibre pull-outs which shows that miscibility between the matrices and fibres was strong. This is obvious from the penetration of the matrices through glass fibres. Additionally, a number of ripple markings and a few abrasion grooves can be observed. Furthermore, by more inspection of the figures, it can be seen that there are some interface gaps between glass fibres and PA6, as seen in the red dashed rectangles marked in Figure 15, Figure 16 and Figure 17. The abrasion direction is indicated by the yellow arrows in the figures. In Figure 14, the worn surface is relatively smooth. Some cavities, grooves, and debris can be seen, which are attributed to the rubbing of the matrix leading to matrix digging out. There was no fibre removal, fibre thinning, or fibre cracking observed, which can be attributed to the low contact pressure being applied to the wear samples. 

Furthermore, the lack of protection and support by glass fibres at high volume fractions, as in Figure 17, led to the exposure of the matrix to more intensive micro-cutting and micro-ploughing attacks by the silicon carbide counter-face. Consequently, more wear of the matrix occurred, leading to an increase in the specific wear rate. The increased amount of wear debris generated due to the abrasion process further reduced the resistance to wear of the thermoplastics rich in fibres owing to the effect of abrasive wear. In 33% glass fibre reinforcement, as seen in Figure 16, crack lines are clearly visible but are not excessively severe, and the fibres are detached from the matrix. In addition, since the load was transferred from polyamide to glass fibres, the wear of polyamide was restrained. This is why incorporating glass fibres enhanced the wear characteristics of polyamide. In 50% glass fibre reinforcement, as seen in Figure 17, it can be seen that rough surfaces with several tearing and branching exist. This tearing appears to originate from the interface between the fibres and the matrix. In pure polyamide, some particles are detached from the body, which leads to the formation of micro-voids. Many micro cracks are observed on the surface of the specimens. More frictional heat is generated between counter-faces in the wear process, and this makes the surface of glass fibre-reinforced polyamide composites soften and become easy to be worn off or be ploughed. It can be seen clearly that there are narrow and obvious grooves, debris, and micro-cracks in Figure 15, Figure 16 and Figure 17, which was dominant in Figure 17, and it suggests that PA650GF suffers the most from abrasive wear. This implies that glass fibres in high volume fraction acted as stress concentrators and promoted the formation of cracks when the samples were loaded. It is possible that increasing the reinforcement content leads to a reduction in the wear properties of neat polyamide 6. In conclusion, 33% fibre volume fraction of reinforcement showed the optimum wear performance among the other volume fractions.

## 4. Comparative Tribological and Mechanical Properties of Reinforced Polymers

**Figure 18:**
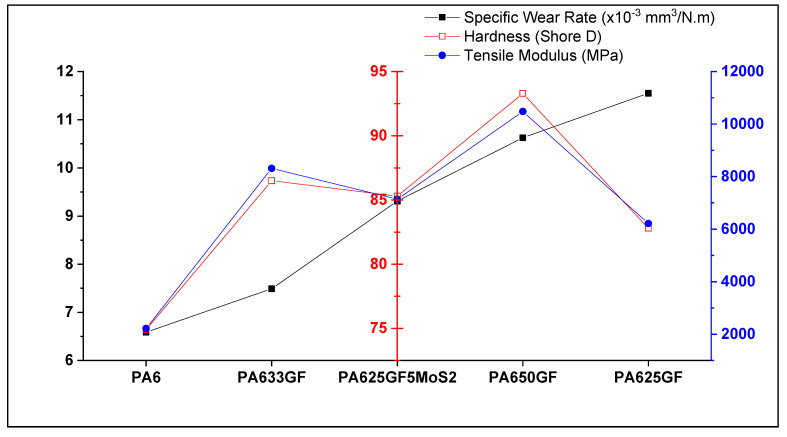
Tensile modulus, hardness, and specific wear rate of PA6 and glass fibre reinforced PA6.

The specific wear rate, Shore D hardness, and tensile modulus of Polyamide 6 and Polyamide 6 composites are shown in Figure 18. It can be observed that for selecting a variation that has high wear resistance, intermediate hardness, and intermediate tensile strength, then the glass fibre-reinforced PA6 at 33% fibre volume fraction would be the optimum solution.

Table 7 shows a summary of the mechanical characteristics of polymers reinforced with either glass fibres or carbon fibres at variable fibre volume fractions. It tabulates values obtained in this study and those reported by other researchers for different materials and processing parameters. From Table 7, it is clear that with increasing the fibre volume fraction, in the polyamide-6 matrix, the tensile strength and compressive strength gradually increase. However, in the present study, the addition of high fibre volume content was negatively affecting the wear resistance of PA6 composites, as the excess amount of fibres acted as a third body during the wear test, which led the material to be suffering aggressively from that abrasive mode. Nevertheless, the addition of 33% of glass fibres was the optimum reinforcing fraction, whereas it showed an improvement in wear resistance when compared with the other fibre volume fractions.

## 5. Conclusions and Closing Remarks

The present research investigates the influence of glass-fibre reinforcements and their role in thermoplastic composite materials for the enhancement of the mechanical performance of thermoplastic composites. Friction and wear are common but critical issues encountered in different engineering sectors that lead to a reduction in machine effectiveness and increased maintenance costs. In summary, the pure thermoplastics and thermoplastic reinforced composites were fabricated by injection moulding technique, and the solid lubricant, molybdenum disulphide, was added to enhance the tribological characteristics of Polyamide 6. According to the different material compositions investigated in the presented research, the main findings can be concluded as follows:

1. The most important conclusion of this study was that there is no correlation between mechanical characteristics with the tribo-performance of thermoplastic composites.
2. The tribo-systems’ responses critically depended on the material design (matrix type and fibre volume fraction).
3. The reinforcement using glass fibres at 33% fibre volume fraction led to superior wear properties among other volume fractions.
4. MoS_2_ improved the load-carrying capacity and thermal stability of glass fibre-reinforced composites since the addition of MoS_2_ as a lubricant significantly reduced the wear rates of PA6 composites by 19.87%.
5. The increase in fibre volume fraction leads to a decrease in the wear resistance of FRPs.

The high potentiality and superiority of fibre-reinforced polymer (FRP) composites have been well displayed in the previous few decades. Therefore, this research identifies the outcomes of developed materials aiming to improve the material design and manufacturing for applications targeting wear and tribological sectors.

## Figures and Tables

**Table 1:** Physical properties of polymeric materials.

Property	Test Method	Material Type	
		Thermoplastic Polyurethane (TPU)	Polyamide 6 (PA6)
Yield Strength (MPa)	ISO 527-2/1A	35.5	77
Flexural Strength (MPa)	ISO 178	-	92
Flexural Modulus (MPa)	ISO 178	-	2500
Notched Izod Impact (kJ/m^2^)	ISO 180	-	3.5
Specific Gravity	ASTM D-792	1.20	1.13
Melting Point (°C)	ISO 3146	-	220

**Table 2:** Designations and compositions of specimens.

Serial	Specimen Code	Chemical Composition		
		Matrix	Reinforcement (% by Volume)	Lubricant
1	PA6	100% PA6	-	-
2	PA625GF	75% PA6	25% Glass Fibre	-
3	PA633GF	67% PA6	33% Glass Fibre	-
4	PA650GF	50% PA6	50% Glass Fibre	-
5	PA625GF5MoS_2_	70% PA6	25% Glass Fibre	5% MoS_2_
6	TPU	100% TPU	-	-

**Table 3:** Mass loss readings of the polymeric and composite samples after wear test.

Sample	Mass Loss (mg)					Mean Value (mg)
PA6	18.00	19.00	19.00	20.00	19.00	19.00
PA633GF	25.00	25.00	26.00	25.00	26.00	25.40
TPU	25.00	25.00	26.00	24.00	28.00	25.60
PA625GF5MoS_2_	34.00	31.00	32.00	30.00	31.00	31.60
PA625GF	39.00	37.00	39.00	38.00	37.00	38.00
PA650GF	37.00	41.00	42.00	45.00	43.00	41.60

**Table 4:** Wear amount and densities of polymeric samples.

Sample	Mass Loss (∆m) [mg]	Density (ρ) [g/cm^3^]	Volume Loss (∆V) [mm^3^]
PA6	19.00	1.128	16.84
TPU	25.67	1.196	21.46

**Table 5:** Wear amount and densities of composite samples.

Sample	Mass Loss (∆m) [mg]	Density (ρ) [g/cm^3^]	Volume Loss (∆V) [mm^3^]
PA633GF	25.33	1.319	19.20
PA625GF5MoS_2_	33.33	1.331	25.04
PA650GF	41.67	1.533	27.18
PA625GF	40.00	1.286	31.10

**Table 6:** Degree of crystallinity of PA6 and PA6 composites.

Serial	Specimen Code	Degree of Crystallinity (Xc%)
1	PA6	47.03
2	PA625GF	40.52
3	PA633GF	37.81
4	PA650GF	33.39
5	PA625GF5MoS_2_	52.38

**Table 7:** Comparative analysis between the results of the current study and the relevant studies from the literature survey.

Material Composition		Tensile Strength	Compressive Strength	Wear Parameters			Specific Wear Rate	Reference
Matrix	Fibre			Applied Load	Distance	Sliding Velocity		
Polyamide-6	-	76.47 MPa	83.275 MPa	10 N	257.48 m	0.31 m/s	6.588 × 10^−3^ mm^3^/Nm	Current study
Polyamide-6	33% vol. glass fibres	286.335 MPa	163.01 MPa	10 N	257.48 m	0.31 m/s	7.491 × 10^−3^ mm^3^/Nm	Current study
Polyamide-6	50% vol. glass fibres	360.782 MPa	228.54 MPa	10 N	257.48 m	0.31 m/s	10.626 × 10^−3^ mm^3^/Nm	Current study
Poly tetra fluoro ethylene/Poly phenylene sulphide	15% vol. carbon fibres	50.2 Mpa	-	200 N	15,000 m	0.42 m/s	2.3 × 10^−6^ mm^3^/Nm	[41]
Epoxy	50% vol. glass fibres	-	-	60 N	5000 m	5.44 m/s	9.2 × 10^−6^ mm^3^/Nm	[42]
Polyamide-6	40% wt. glass fibres	192 Mpa	-	10 N	1000 m	1.6 m/s	0.23 × 10^−7^ mm^3^/N	[43]
Polyamide-6	40% wt. glass fibres	192 Mpa	-	10 N	1000 m	4.0 m/s	0.26 × 10^−7^ mm^3^/Nm	[43]
Epoxy	15% vol. carbon fibres	-	-	40 N	72,000 m	1.0 m/s	16 × 10^−7^ mm^3^/Nm	[44]
Polyamide-6	10% wt. glass fibres	53.63 Mpa	-	15 N	1000 m	2.0 m/s	3.0 × 10^−3^ mm^3^/Nm	[45]
Polyamide-6	30% wt. glass fibres	86.01 Mpa	-	15 N	1000 m	1.0 m/s	1.38 × 10^−4^ mm^3^/Nm	[45]
Polyamide 66	30% vol. glass fibres	140.03 Mpa	-	200 N	3024 m	0.42 m/s	25.3 × 10^−6^ mm^3^/Nm	[46]
Polyamide	11.3% vol. glass fibres	-	-	19.6 N	15,000 m	1.0 m/s	2.87 × 10^−6^ mm^3^/Nm	[47]
Polyamide	20.7% vol. glass fibres	-	-	19.6 N	30,000 m	1.0 m/s	1.66 × 10^−6^ mm^3^/Nm	[47]
Polyamide-6	20% wt. glass fibres	-	-	40 N	1800 m	1500 rpm	12.0 × 10^−7^ mm^3^/Nm	[48]
Polyamide-6	20% wt. glass fibres	-	-	160 N	1800 m	1500 rpm	8.0 × 10^−7^ mm^3^/Nm	[48]

## Data Availability

The raw/processed data required to reproduce these findings cannot be shared at this time as the data also forms part of an ongoing study.

## Nomenclature

ASTM	American Society for Testing and Materials
CF	Carbon Fibre
FESEM	Field Emission Scanning Electron Microscopy
FRP	Fibre Reinforced Polymer
GF	Glass Fibre
GFR	Glass Fibre Reinforced
Micro-CT	Micro-Computed Tomography
MoS_2_	Molybdenum Disulphide
PA6	Polyamide 6
PTFE	Polytetrafluoroethylene
SiC	Silicon Carbide
TiO_2_	Titanium Dioxide
TPU	Thermoplastic Polyurethane
W_s_	Specific Wear Rate
X_c_	Degree of Crystallinity
